# Development of a measurement tool to assess public awareness of cancer

**DOI:** 10.1038/sj.bjc.6605385

**Published:** 2009-12-03

**Authors:** S Stubbings, K Robb, J Waller, A Ramirez, J Austoker, U Macleod, S Hiom, J Wardle

**Affiliations:** 1 Cancer Research UK Health Behaviour Research Centre, Department of Epidemiology and Public Health, UCL, Gower Street, London WC1E 6BT, UK; 2 Cancer Research UK Promoting Early Presentation Group, Institute of Psychiatry, King's College London, St Thomas' Hospital, London, UK; 3 Cancer Research UK Primary Care Education Research Group, Cancer Epidemiology Unit, University of Oxford, Richard Doll Building, Roosevelt Drive, Oxford, UK; 4 General Practice and Primary Care, Division of Community Based Sciences, Faculty of Medicine, University of Glasgow, 1 Horselethill Road, Glasgow, UK; 5 Health Information Department, Cancer Research UK, 61 Lincoln's Inn Fields, London, UK

**Keywords:** cancer awareness, measurement, psychometrics

## Abstract

**Objective:**

We aimed to develop and validate a measurement tool to assess cancer awareness in the general population: the cancer awareness measure (CAM).

**Methods:**

Items assessing awareness of cancer warning signs, risk factors, incidence, screening programmes and attitudes towards help seeking were extracted from the literature or generated by expert groups. To determine reliability, the CAM was administered to a university participant panel (*n*=148), with a sub-sample (*n*=94) completing it again 2 weeks later. To establish construct validity, CAM scores of cancer experts (*n*=12) were compared with those of non-medical academics (*n*=21). Finally, university students (*n*=49) were randomly assigned to read either a cancer information leaflet or a leaflet with control information before completing the measure, to ensure the CAM was sensitive to change.

**Results:**

Cognitive interviewing indicated that the CAM was being interpreted as intended. Internal reliability (Cronbach's *α*=0.77) and test–retest reliability (*r*=0.81) were high. Scores for cancer experts were significantly higher than those for non-medical academics (*t*(31)=6.8, *P*<0.001). CAM scores were higher among students who received an intervention leaflet than the control leaflet (*t*(47)=4.8, *P*<0.001).

**Conclusions:**

These studies show the psychometric properties of the CAM and support its validity as a measure of cancer awareness in the general population.

Cancer is a major burden worldwide (Parkin *et al*, 2005) and a leading cause of mortality in the United Kingdom, claiming over 150 000 lives a year (Cancer Research UK, 2009). Around 289 000 new cases are diagnosed annually in the United Kingdom, with one in three people developing cancer in their lifetime (Cancer Research UK, 2008a). Despite progress in reducing mortality rates, changes in the age distribution of the population will mean that cancer incidence will continue to rise (Boyle and Ferlay, 2005). In recent years, the UK Governments have developed strategies aimed at reducing cancer incidence and mortality; the most recent for England is the NHS Cancer Reform Strategy (Department of Health, 2007), which emphasises the importance of raising public awareness of early warning signs and risk factors.

Existing evidence indicates that public awareness of warning signs is poor. In a population-based survey in England, fewer than 1 in 10 respondents could recall Europe Against Cancer's seven warning signs for cancer (Brunswick *et al*, 2001). These findings are not unique to this study nor limited to generic warning signs; awareness is also low for a range of cancers (Wardle *et al*, 2001; Grunfeld *et al*, 2002; McCaffery *et al*, 2003; Rudberg *et al*, 2005; West *et al*, 2006; FitzGerald *et al*, 2008).

European comparisons show the United Kingdom to have lower than average cancer survival rates (Coleman *et al*, 2003), part of which is due to patients in the United Kingdom having more advanced stage of disease at diagnosis. Later presentation of cancer symptoms in the United Kingdom than in other European countries may contribute to this. Raising public awareness of warning signs and promoting prompt presentation could reduce patient-attributable delay and result in diagnosis at an earlier stage (Ramirez *et al*, 1999; Richards *et al*, 1999; Macdonald *et al*, 2006).

Awareness of cancer risk factors except for tobacco use is also low (Wardle *et al*, 2001; Grunfeld *et al*, 2002; McCaffery *et al*, 2003; Marlow *et al*, 2007; Redeker *et al*, 2009), particularly in relation to weight, alcohol consumption, high-fat diet, exercise and older age. It is estimated that around half of all cancers could be prevented by modification of lifestyle risk factors (Cancer Research UK, 2008b). Although awareness alone may not be sufficient to motivate change, it is unrealistic to expect changes in behaviour if people are not at least aware of the risk factors (Viswanath *et al*, 2006).

There is currently no validated measure of general public awareness of cancer, although several questionnaires have been developed to assess awareness of specific cancers (Stager, 1993; Rees *et al*, 2003; Green and Kelly, 2004). In a review of the literature, Adlard and Hume (2003) also identified lack of agreement over the best way to measure cancer awareness. As a result, different question formats are often used. Questions asked in a prompted (recognition) format can elicit higher apparent levels of cancer awareness than those asked in an unprompted (recall) format (Waller *et al*, 2004). Such variations in responses make it difficult to establish current levels of awareness or make comparisons across studies.

Education campaigns designed to improve awareness of cancer in the general population have been carried out, but without validated instruments, it is difficult to evaluate effectiveness. This highlights the need for a measure that will enable both researchers and campaigning groups to evaluate the impact of their activities.

The aim of this research was therefore to develop and validate a standardised measurement tool to assess cancer awareness. A good measure should have face validity, the questions should be interpreted as intended by the target audience, and it should give stable results across two occasions between which knowledge has not changed (test–retest reliability). Good reliability is essential for obtaining precise estimates of knowledge and for giving the best statistical power to detect change. A good measure also needs to be valid, that is, groups who by general consensus have a higher standing on the relevant constructs should score higher; in this case, we compared cancer experts and equivalently educated non-experts to test the instrument's sensitivity to knowledge. Finally, if it is to have value in assessing the impact of public health interventions, it should be sensitive to change; we tested this by comparing scores before and after exposure to a brief educational intervention.

## Materials and methods

### Generation of items

Following a review of the literature, existing awareness questionnaires were examined and relevant items extracted. This was supplemented with a review of the ‘grey’ literature (i.e. unpublished surveys carried out by cancer charities and other organisations) to include items not published in academic journals. Following this review, an item pool consisting of 137 items was created. These covered a range of topics including awareness of warning signs and risk factors, cancer incidence and awareness of national screening programmes. Items were then excluded if they were poorly worded, used terminology not frequently used in the United Kingdom (e.g. Pap test) or were attitudinal in nature (e.g. ‘I believe there are no early symptoms of cancer’). Items relating to awareness of the purpose of screening, the benefits of early detection and cancer survival rates were also omitted from the measure because the primary focus was symptom recognition. In addition, the research team generated several items specifically for the instrument that had not been used in previous questionnaires.

Once consensus over the items had been reached, a first version of the cancer awareness measure (CAM) was circulated to a panel of experts (*n*=16) including academic researchers, cancer charity representatives, general practitioners, oncologists and experts in the field of questionnaire design, to ensure content validity and face validity. In addition, cognitive interviews were conducted with the general public. These encourage respondents to verbalise their cognitions, making it possible to identify areas where interpretation of the questions is ambiguous (Collins, 2003). Cognitive interviews were conducted with a small sample of participants (*n*=6) aged between 23 and 70 years. Minor modifications were made to the phrasing of several items as a result.

The final version of the CAM consisted of the following: (i) 10 items on awareness of warning signs (one open-ended question and nine recognition items); (ii) nine items on anticipated time to seek medical advice (asking about each of the warning signs); (iii) 10 items on barriers to seeking medical advice (covering a range of practical, service delivery and emotional barriers); (iv) 13 items on awareness of risk factors (one open-ended question, 11 recognition items and one asking participants to rank the importance of different types of risk factor); (v) seven items on cancer incidence (one asking about overall cancer incidence and six asking about the three most common cancers for men and women) and (vi) six items on awareness of NHS screening programmes (asking about awareness of the cervical, breast and bowel screening programmes and the age from which screening is offered for each).

### Validating the CAM

Validating the CAM was a three-stage process, with each stage assessing a different aspect of reliability or validity. The aims of stage one were to establish internal reliability and test–retest reliability and carry out item analyses; stage two was designed to establish construct validity and stage three to ensure that the measure was sensitive to increases in levels of awareness.

Data were analysed using SPSS 14.0. Parametric statistics (e.g. Pearson's correlation, *t*-tests) were performed to analyse the reliability and validity of the measure. Descriptive statistics were used to examine the characteristics of the samples.

## Results

### Stage 1: Internal reliability and test–retest reliability

#### Sample and methods

Five hundred and fifty-one e-mails were sent to a research ‘participant panel’ with an invitation to complete the CAM anonymously online. The panel consisted of members of the general public who had previously indicated that they were willing to participate in research. The questionnaire was completed in the available time by 148 (27%) panel members. The majority of respondents were women (76%), white (86%) and educated to degree level (68%) (Table 1). Two weeks later, respondents were asked to complete the CAM a second time. Two weeks was judged as an adequate period of time for respondents neither to recall precisely their original answers, nor to be likely to have had any major changes in cancer awareness. Ninety-four participants (63%) completed the questionnaire again. Data were matched using e-mail addresses.

Internal reliability assesses the extent to which all the questionnaire items measure the same underlying construct (Kline, 2000). It is assessed using Cronbach's *α* and a minimum score of 0.7 should be obtained for a questionnaire to be considered reliable (Bland and Altman, 1997). To assess the stability of a questionnaire over time, a measure of test–retest reliability must be calculated (Kline, 2000). Pearson's correlations are computed using scores from two time points. It is important to identify whether respondents find items too easy or too difficult to answer, and it is recommended that items are excluded if they are answered correctly by >80% or <20% of participants (Kline, 2000). Item discrimination reveals the ability of an individual item to discriminate between those who have high or low overall knowledge scores, and items should be discarded if an item-to-total correlation of <0.2 is yielded (Streiner and Norman, 1995).

### Analysis and results

#### Internal reliability

A Cronbach's *α* of 0.77 was achieved for the whole questionnaire, with the following *α* values obtained for each sub-section: warning signs (9 recognition items) 0.77; anticipated time to seek medical advice (9 items) 0.90; barriers to seeking medical advice (10 items) 0.73; risk factors (11 recognition items) 0.79; NHS screening programmes (6 items) 0.54. Despite obtaining an *α* below the minimum cutoff, the decision was made to retain awareness of NHS screening programmes on the grounds of content validity.

#### Test–retest reliability

With the exception of incidence of common cancers, high correlations over time were found for all sections (Table 2), all of which reached statistical significance (*P*<0.001).

#### Item difficulty

The majority of items matched the criterion of being answered correctly by <80% and >20% of participants. The few that did not were retained on the basis of face validity (e.g. smoking being a risk factor for cancer, a lump being a warning sign for cancer), because respondents might be surprised if they were not included.

#### Item discrimination

Analyses revealed item-to-total correlations >0.2 for each item, suggesting all items in the CAM should be retained.

### Stage 2: Construct validity: cancer ‘experts’ *vs* non-medical academics

#### Sample and methods

The ‘known-groups’ method was used to establish construct validity. If the scores of two groups known to differ in levels of cancer awareness are significantly different then the validity of the questionnaire is supported (DeVellis, 2003). Cancer experts (*n*=12) were recruited from a large cancer charity, while the ‘non-expert’ group comprised non-medical academics (*n*=21) recruited from a range of departments in the university. Participants were invited to take part by e-mail and could either complete the questionnaire electronically or print it out and complete a paper copy. The demographic characteristics of the samples can be seen in Table 3.

### Analyses and results

The cancer experts scored consistently higher than the non-medical academics (Table 4) and, with the exception of incidence, this reached statistical significance for each awareness section. Although differences in incidence scores were not statistically significant, the standard deviations varied considerably (0.8 for the cancer experts and 12.5 for the non-medical group), suggesting that experts were more consistent at answering the questions.

### Stage 3: Sensitivity to change: brief educational intervention

#### Sample and methods

A convenience sample of 49 undergraduate and postgraduate students was recruited to participate in this stage. They were randomised to receive one of two leaflets to read before completing a paper copy of the CAM. One was an educational leaflet (‘Cancer: the facts’), which included information on the aetiology of cancer, incidence rates, warning signs, risk factors and available NHS screening programmes. The other was a control leaflet (‘Recycle to save the environment’).

Demographic characteristics of the two groups were similar (Table 5), with no significant differences in age, gender or ethnic origin being observed.

### Analyses and results

The cancer education group scored consistently higher than the control group and, excluding results for cancer incidence, this reached statistical significance for all awareness sections (Table 6). Not surprisingly for such a brief intervention there was comparatively small mean difference between the education and control groups, but there was also differentiated variability with standard deviations of 3.4 (education) and 11.5 (control).

## Discussion

There has been a good deal of interest in public awareness of cancer, but in most studies measures are developed on an *ad hoc* basis, often with a specific population in mind, and rarely address psychometric properties. This not only limits generalisation to other groups, but also precludes comparisons between studies or groups.

The CAM was developed to provide a validated measure of awareness of early warning signs and risk factors, and barriers to seeking medical advice. Reliability of the measure was high, with a total Cronbach's *α* of 0.77 and all but one of the sub-scales reaching the recommended cutoff of 0.7. Test–retest reliability was also good, with all sub-scales except awareness of the incidence of common cancers being >0.7. The low reliability of the incidence items probably reflects the fact that people had little idea and were just guessing the answers; and not making the same guess on different occasions. This is understandable because although people may be able to identify the most common cancer in men (prostate) and women (breast), the second and third most common cancers are rarely publicised as such. However, given that awareness of the most common cancers could improve, these items were retained.

Construct validity was established using a ‘known-groups’ design. Overall, those with expertise in cancer achieved significantly higher mean scores than non-medical academics, showing the CAM has the ability to distinguish between groups with established differences in levels of awareness. In addition, the CAM was shown to be sensitive to increases in awareness following a brief educational intervention.

### Limitations

The limitations to the validation of the measure should be noted. The mode of administration varied across different stages of piloting, including online and with paper-and-pencil versions. Concerns have been raised about the quality of survey data collected through different modes (Bowling, 2005), although the mode of administration was consistent within each stage of piloting so the potential impact would have been kept to a minimum. For use in population surveys, we suggest that the CAM should ideally be given in an interview format to prevent participants from changing their previous answers in response to the prompted format of subsequent questions. The pilot samples tended to be female and educated to degree level, which limits generalisation to other populations. However, given that the CAM was sensitive to differences in levels of awareness in a highly educated academic sample, it is likely that differences would be equally marked in less educated populations. To date, the CAM is only available in English, but we anticipate that it will be translated into a range of languages and available for use in the future.

### Future work

This paper describes the development of the generic CAM. Work is now underway to develop tumour-specific versions of the CAM, including breast, prostate, bowel, cervical and ovarian, and more tumour-specific versions are planned. It is anticipated that the CAM will evolve over time to reflect advances in knowledge, and to include items on cancer beliefs that may help us to better understand cancer-related behaviour.

### CONCLUSION

The CAM is a reliable and valid measure of cancer awareness and can be used to provide a comprehensive assessment of cancer awareness. The CAM has now been administered in a large-scale, population-based, British sample with a substantial ethnic boost sample, using a face-to-face, home-based interview methodology, so standardised population data for the United Kingdom are available for reference (Robb *et al*; Waller *et al*). In addition, the CAM can be used by researchers to develop informed interventions and to assess the impact of interventions designed to target gaps in public awareness of cancer either in whole populations or specific sub-groups.

## Figures and Tables

**Table 1:** Demographic characteristics of the online study sample (*n*=148)

	***N***	**%^a^**
*Gender*		
Male	36	24.3
Female	112	75.7
		
*Age (years)*		
18–24	40	27.0
25–34	33	22.3
35–44	9	6.1
45–54	11	7.4
55–64	39	26.4
65 and over	16	10.8
		
*Ethnic origin*		
White	126	85.1
Other	20	13.5
		
*Employment status*		
Employed full-time	37	25.0
Employed part-time	11	7.4
Student	62	41.9
Other	38	25.7
		
*Highest qualification obtained*		
No qualifications	2	1.4
O level/GCSE	5	3.4
A level	14	9.5
Degree or above	101	68.2
Still studying	23	15.5
		
*Marital status*		
Single	58	39.2
Married/cohabiting	75	50.7
Divorced/widowed	14	9.5

a Some variables do not add up to 100% due to missing data.

**Table 2:** Test–retest reliability of the CAM (*n*=94)

**Awareness section**	**Test–retest reliability**
Warning signs	0.73
Anticipated time to seek advice	0.86
Barriers to seeking advice	0.72
Risk factors	0.73
Incidence per 100 people	0.78
Incidence – common cancers	0.33
Screening programmes	0.75
Screening programmes – age at first invitation	0.77
Total	0.81

Abbreviation: CAM=cancer awareness measure.

**Table 3:** Demographic characteristics of the two samples (*n*=33)

	**Non-medical academics (*n*=21)**		**Cancer experts (*n*=12)**	
	***n***	**%**	***n***	**%**
*Age (years)*				
25–34	2	9.5	7	58.3
35–44	8	38.1	4	33.3
45–54	9	42.9	—	—
55–64	2	9.5	1	8.3
				
*Gender*				
Male	9	42.9	1	8.3
Female	12	57.1	11	91.7
				
*Highest qualification*				
Degree	—	—	2	16.7
Masters	2	9.5	3	25.0
PhD or equivalent	19	90.5	5	41.7
Other	—	—	2	16.7

**Table 4:** Differences in awareness scores between non-medical academics and cancer experts (*n*=33)

**Awareness section**	**Non-medical academics (*n*=21)**		**Cancer experts (*n*=12)**		
**(max score)**	**Mean**	**s.d.**	**Mean**	**s.d.**	***t* (31)**
Warning signs (9)	7.3	2.4	8.8	0.4	2.9^*^
Risk factors (55)	42.3	4.1	50.8	4.7	5.4^**^
Incidence per 100 people^a^	34.0	12.5	33.3	0.8	−0.2
Incidence – common cancers (6)	2.3	1.2	3.5	1.4	2.7^*^
Screening programmes (3)	1.9	0.4	3.0	0.0	9.4^**^
Screening programmes – age at first invitation (3)	0.7	0.6	2.2	1.1	4.2^**^
Total (77)	57.2	5.9	69.3	5.6	6.8^**^

^*^P<0.05.

^**^P<0.001.

a Participants were given a score of ‘1’ if their answer was correct (correct range, 30–36).

**Table 5:** Demographic characteristics of the two samples (*n*=49)

	**Control (*n*=24)**		**Intervention (*n*=25)**	
	***n***	**%**	***n***	**%**
*Age (years)*				
18–24	9	62.5	12	48.0
25–34	15	37.5	12	48.0
35+	—	—	1	4.0
				
*Gender*				
Male	9	37.5	9	36.0
Female	15	62.5	16	64.0
				
*Ethnic origin*				
White	17	70.8	16	64.0
Other	7	29.2	9	36.0

**Table 6:** Differences in awareness scores between control and intervention participants (*n*=49)

**Awareness section**	**Control (*n*=24)**		**Intervention (*n*=25)**		
**(max score)**	**Mean**	**s.d.**	**Mean**	**s.d.**	***t* (47)**
Warning signs (9)	6.0	1.9	7.3	0.9	2.7^*^
Risk factors (55)	39.8	4.6	43.7	5.0	2.8^*^
Incidence per 100 people^a^	29.2	11.5	32.8	3.4	1.5
Screening programmes (3)	1.8	0.7	2.7	0.7	4.2^**^
Screening programmes – age at first invitation (3)	0.6	0.6	1.8	0.9	5.2^**^
Total (71)^b^	48.7	5.8	56.3	5.7	4.6^**^

^*^P<0.05.

^**^P<0.001.

a Participants were given a score of ‘1’ if their answer was correct (correct range, 30–36).

b This total score does not include all components of the awareness measure. The intervention leaflet did not contain information on timely presentation, barriers to seeking medical advice or incidence of most common cancers, so these items have been omitted from the analysis.
